# Exchange of a Single Amino Acid Residue in the HybG Chaperone Allows Maturation of All H_2_-Activating [NiFe]-Hydrogenases in *Escherichia coli*

**DOI:** 10.3389/fmicb.2022.872581

**Published:** 2022-03-29

**Authors:** Alexander Haase, R. Gary Sawers

**Affiliations:** Institute of Microbiology, Martin-Luther-University Halle-Wittenberg, Halle, Germany

**Keywords:** hydrogen evolution, hydrogen oxidation, HypC chaperone, HybG chaperone, HypD, maturation, NiFe-hydrogenase

## Abstract

The biosynthesis of the NiFe(CN)_2_CO organometallic cofactor of [NiFe]-hydrogenase (Hyd) involves several discreet steps, including the synthesis of the Fe(CN)_2_CO group on a HypD-HypC scaffold complex. HypC has an additional function in transferring the Fe(CN)_2_CO group to the apo-precursor of the Hyd catalytic subunit. Bacteria that synthesize more than one Hyd enzyme often have additional HypC-type chaperones specific for each precursor. The specificity determinants of this large chaperone family are not understood. *Escherichia coli* synthesizes two HypC paralogs, HypC and HybG. HypC delivers the Fe(CN)_2_CO group to pre-HycE, the precursor of the H_2_-evolving Hyd-3 enzyme, while HybG transfers the group to the pre-HybC of the H_2_-oxidizing Hyd-2 enzyme. We could show that a conserved histidine residue around the amino acid position 50 in both HypC and HybG, when exchanged for an alanine, resulted in a severe reduction in the activity of its cognate Hyd enzyme. This reduction in enzyme activity proved to be due to the impaired ability of the chaperones to interact with HypD. Surprisingly, and only in the case of the HybG_*H*52*A*_ variant, its co-synthesis with HypD improved its interaction with pre-HycE, resulting in the maturation of Hyd-3. This study demonstrates that the conserved histidine residue helps enhance the interaction of the chaperone with HypD, but additionally, and in *E. coli* only for HybG, acts as a determinant to prevent the inadvertent maturation of the wrong large-subunit precursor. This study identifies a new level of control exerted by a bacterium synthesizing multiple [NiFe]-Hyd to ensure the correct enzyme is matured only under the appropriate physiological conditions.

## Introduction

**FIGURE 1:**
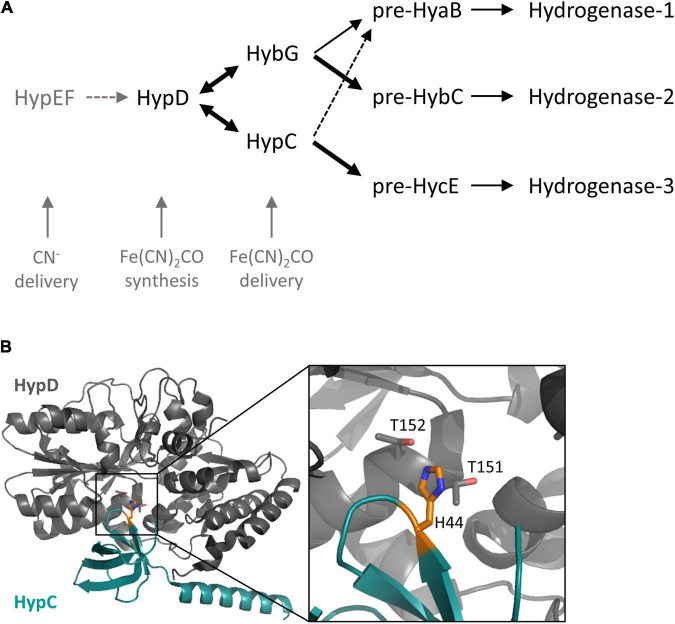
The HypC chaperones involved in the maturation of hydrogenases in *E. coli*. **(A)** Schematic overview of the roles of HypC and HybG and their respective precursor specificities. The thickness of the arrow correlates with precursor preference. Dotted arrows indicate weak interaction, while double-headed arrows signify a reversible interaction. **(B)** Close-up of the location of the conserved histidine at the interface between HypC and HypD in the structure of scaffold complex determined for *Thermococcus kodakarensis* (PDB structure 3VYR; Watanabe et al., 2012). Note that the original residue numbering of HypD_*Tk*_ and HypC_*Tk*_ was retained in the structural analysis, so histidine 44 corresponds to H51 of *Escherichia coli* HypC, and threonine 151 and 152 of HypD_*Tk*_ correspond to threonine T149 and T150 in HypD from *E. coli*. The location of the conserved histidine is indicated in orange and the structural representation was determined using PyMOL (The PyMOL Molecular Graphics System, version 2.5, Schrödinger, LLC).

During growth under anoxic conditions, *Escherichia coli* synthesizes three comparatively abundant [NiFe]-hydrogenases (Hyd) (Pinske and Sawers, 2016; Sargent, 2016). Of these three enzymes, two, Hyd-1 and Hyd-2, are principally functional in H_2_ oxidation (Ballantine and Boxer, 1985; Sawers et al., 1985), while the third, Hyd-3, is a component of the H_2_-evolving formate hydrogenlyase (FHL) complex (McDowall et al., 2014). All three enzymes have the same organometallic cofactor [NiFe(CN)_2_CO, or [NiFe]-cofactor] in their active site, which is required for reversible catalytic dihydrogen (H_2_) activation by the enzyme (Böck et al., 2006; Lacasse and Zamble, 2016). The biosynthesis and insertion of this [NiFe]-cofactor into the large-subunit precursors of the respective Hyd enzymes requires the combined actions of six accessory proteins, the functions of which have been described previously (Böck et al., 2006). While the synthesis of this [NiFe]-cofactor is not the focus of this study, it is nevertheless important to stress that the Fe(CN)_2_CO component of the cofactor is assembled on a separate scaffold complex comprising the iron-sulfur protein HypD and either of the two small ∼10 kDa proteins called HypC or HybG. These latter two paralogs, which are the focus of this study, are structurally and functionally related and appear to have multifarious functions, being required for both biosynthesis and delivery of the Fe(CN)_2_CO group to the Hyd large-subunit precursors (Blokesch et al., 2001; Böck et al., 2006). They concomitantly acquire the iron ion and a CO_2_ molecule, which potentially acts as a source of the CO ligand (Soboh et al., 2013; Arlt et al., 2021); the cellular sources of these are unknown. In complex with HypD, either HypC or HybG is required to aid the synthesis of the Fe(CN)_2_CO group (Blokesch et al., 2002, 2004). Once the synthesis of this group has been completed, the chaperones deliver Fe(CN)_2_CO to their designated large-subunit precursors (Arlt et al., 2021). The roles of HypC and HybG are schematically summarized in Figure 1A. Importantly, HypC specifically delivers the Fe(CN)_2_CO group to pre-HycE, the precursor of Hyd-3, and to a lesser extent to pre-HyaB, the precursor of Hyd-1, while HybG delivers the group specifically and preferentially to pre-HybC, and to pre-HyaB (Blokesch et al., 2001). The aim of this study is, therefore, to provide further insight into how the precursor specificity of the chaperones is delimited.

A recent study has demonstrated that HybG interacts with either HypD or pre-HybC, presumably shuttling Fe(CN)_2_CO from the HypD to pre-HybC (Arlt et al., 2021). These findings corroborate earlier proposals that the distinct precursor specificity shown by HybG and HypC for particular client proteins, as well as a common specificity for HypD, signifies a chaperone function for these small proteins (Drapal and Böck, 1998; Wolf et al., 1998; Jones et al., 2004). Because these proteins are small, it remains unclear whether common determinants on HypC and HybG govern their interaction with different precursors and with HypD, or whether different motifs play a role. Importantly, what prevents HybG from interacting with pre-HycE and activating Hyd-3, but allows it to interact with pre-HybC and pre-HyaB?

One answer to this question likely lies in the fact that the gene encoding HybG is located within the *hyb* structural gene operon, which encodes Hyd-2 (Menon et al., 1994). Typically, genes encoding HypC family members are located adjacent to those encoding HypD (Böck et al., 2006; Lacasse and Zamble, 2016), so the physical separation of the *hybG* gene from *hypD* on the *E. coli* genome presumably helps to limit HybG’s ability to compete with HypC for HypD interaction.

Due to the lack of structural information on the HybG-pre-HybC or HypC-pre-HycE complexes, we must rely on the knowledge gained from *in vivo* studies performed with *E. coli* (Hartwig et al., 2015; Thomas et al., 2018), and from structural analyses of HypC and HypD from *Thermococcus kodakarensis* (Watanabe et al., 2007, 2012), to gain insight into potentially key residues or motifs involved in distinguishing client proteins.

The structure of HypC has revealed that, along with the essential *N*-terminal cysteine residue (C2), there is a highly conserved histidine residue at amino acid position 51 (H51; *E. coli* numbering) (Figure 1B), which is proximal to C2, and consequently has been suggested to have a role in stabilizing the binding of the Fe(CN)_2_CO group (Watanabe et al., 2007). The limited *in vivo* studies examining the role of this histidine residue have reported that its exchange for arginine in HypC essentially abolishes the Hyd-3-dependent H_2_ evolution (Blokesch, 2004), while an *in vitro* study performed with the same variant reported that it was impaired in cofactor synthesis (Soboh et al., 2013). The examination of the crystal structure of the HypCD complex from *T. kodakarensis* located it to the interaction surface between the proteins (Watanabe et al., 2012; Figure 1B), which might also explain the biosynthesis-deficient phenotype observed when it is exchanged for another residue (Soboh et al., 2013).

In an attempt to resolve the function of this residue in the chaperone we have used HypC and HybG of *E. coli* as a model system and made a new variant by exchanging this residue for alanine in both proteins (position 52 in HybG). Using combined *in vitro* and *in vivo* experimental approaches, we show that this conserved histidine residue is indeed important to allow HypC and HybG to interact effectively with HypD. Unexpectedly, however, our studies also revealed that the HybG_*H*52*A*_ variant gains the ability to interact with pre-HycE, thus identifying this histidine residue as a key determinant in ensuring that HybG normally does not mature Hyd-3 *in vivo*.

## Materials and Methods

Bacterial strains and plasmids. The *E. coli* strains used in this study included MC4100 *[* F–, *araD139*, ***Δ*** (*argF-lac*)*U169*, λ*^–^*, *rpsL150*, *relA1*, *deoC1*, *flhD5301*, ***Δ*** (*fruK-yeiR*)*725*(*fruA25*), *rbsR22*, ***Δ*** (*fimB-fimE*) (Casadaban, 1976)*]*, and its isogenic mutant derivative SHH228 (like MC4100, but Δ*hypC* Δ*hybG*) (Hartwig et al., 2015). All of the plasmids used in this study are listed in Table 1 and were introduced individually into strain SHH228 for either determination of Hyd-associated enzyme activities or protein purification. To construct plasmid phypCstrep carrying the native *hypC* gene with an additional *C*-terminal Strep-tag II coding sequence, genomic DNA isolated from *E. coli* strain MC4100 was used as a template for the PCR-amplification of the *hypC* gene using oligonucleotide primers hypC_*Bsa*I_IBA3_fwd (5′-GCACACGGTCTCAAATGTGCATAGGCGTTCCCGG-3′) and hypC_*Bsa*I_IBA3_rev (5′-GCACACGGTCTCAGCGCTGACATCCGGCTCAACGTCAAA-3′). The PCR product was digested with *Bsa*I and the resulting DNA fragment was ligated into the *Bsa*I-digested pASK-IBA3plus vector (IBA Lifesciences, Göttingen, Germany) to generate the plasmid phypCstrep. The plasmids phypC(H51A)strep, pT-hypDC(H51A)Strep, phybG(H52A)strep, and pT-hybG(H52A)-hypDEF were generated by PCR-based site-directed mutagenesis using the Q5® Site-Directed Mutagenesis Kit (New England Biolabs, United States). Plasmids phypC(H51A)strep and pT-hypDC(H51A)Strep were generated by changing codon 51 in the *hypC* gene on the plasmids phypCstrep and pT7-hypDCStrep (Blokesch et al., 2004), respectively, from CAC to GCC employing the oligonucleotides hypC_H51A_fw (5′-GGTACTGGTAGCCGTTGGCTTTGCCAT-3′) and hypC_H51A_rv (5′-CACTGGCCCACGCGCGGC-3′). Plasmids phybG(H52A)strep and pT7-hybG(H52A)-hypDEF were generated by changing codon 52 in the *hybG* gene on the plasmids phybGstrep (Soboh et al., 2014) and pT-hybG-hypDEF (Soboh et al., 2014), respectively, from CAT to GCC using the oligonucleotides hybG_H52A_fw (5′- GGTGCTGGTAGCCGTCGGATTTGC-3′) and hybG_H52A_rv (5′- CACTGGCCCAGTAGATCG-3′).

**TABLE 1:** Strains and plasmids used in this study.

Strain or plasmid	Relevant genotype or characteristic(s)	References
Strains		
MC4100	F^–^ *araD139* (*argF-lac*)*U169 ptsF25 deoC1 relA1 flbB5301 rspL150*	Casadaban, 1976
SHH228	Like MC4100, but Δ*hypC* Δ*hybG*	Hartwig et al., 2015
Plasmids		
pASK-IBA3		
phypCstrep	pASK-IBA3, *hypC* with C-terminal Strep-tag II, Amp*^R^*	This study
phypC(H51A)strep	Like phypCstrep, but codon 51 in *hypC* changed CAC → GCC, Amp*^R^*	This study
pT-hypDCStrep	pT7-7, *hypD, hypCStrep*, Amp*^R^*	Blokesch et al., 2004
pT-hypDC(H51A)Strep	Like pT-hypCDStrep, but codon 51 in *hypC* changed CAC → GCC, Amp*^R^*	This study
phybGstrep	pASK-IBA3, *hybG* with C-terminal Strep-tag II, Amp*^R^*	Soboh et al., 2014
phybG(H52A)strep	Like phybGstrep, but codon 52 in *hybG* CAT → GCC, Amp*^R^*	This study
*^a^*pT-hybG-hypDEF	pT7-7, *hypD, hypE*, *hybGStrep*, *hypF*, Amp*^R^*	Soboh et al., 2014
pT-hybG(H52A)-hypDEF	Like pT-hybG-hypDEF, but codon 52 in *hybG* CAT → GCC, Amp*^R^*	This study
*^b^*pHycEH	pACYC-Duet1, MCS1:_*His*_*hycE* (internal His-tag on HycE), MCS2;_*Strep*_*hycH* (*N*-terminal Strep-tag II on HycH), Cm*^R^*	Lindenstrauß et al., 2017

*^a^Note that the presence of the hypE and hypF genes had no influence on the function of HybG.*

*^b^The vector was used as a resource to purify His-tagged pre-HycE separately from Strep-tagged HycH.*

### Growth Conditions

In preparation for routine microbiology and molecular biology experiments, such as cloning, strains were grown on LB-agar plates or in LB-broth at 37°C (Miller, 1972). Anaerobic growth for Hyd enzyme assays and enzyme activity-staining after native polyacrylamide gel electrophoresis (PAGE) was performed at 37°C as standing liquid cultures in the buffered rich medium TGYEP (1% w/v tryptone, 0.5% w/v yeast extract, 0.8% w/v glucose, 100 mM potassium phosphate, pH 6.5) (Begg et al., 1977). The growth medium was supplemented with trace element solution SLA (Hormann and Andreesen, 1989). When required, the antibiotics ampicillin, chloramphenicol, or kanamycin were added to a final concentration of 100, 25, or 50 μg ml^–1^, respectively. Cells were harvested anaerobically when cultures had reached an OD_600_ nm of between 0.8 and 1.2 by centrifugation at 5,000 *g* for 15 min at 4°C. Cell pellets were either used immediately or stored at –20°C until use.

For anaerobic protein overproduction experiments, *E. coli* strain SHH228 (Δ*hypC* Δ*hybG*) was transformed with the indicated plasmids using standard procedures (Sambrook et al., 1989). Cultivation of cells was performed in modified TB medium (2.4% w/v yeast extract, 1.2% w/v peptone from casein, 0.04% w/v glycerol, 0.4% w/v glucose and 0.003% w/v magnesium sulfate heptahydrate) (Soboh et al., 2012). Depending on the plasmid used, the medium also included either 100 μg ml^–1^ ampicillin or 15 μg ml^–1^ chloramphenicol to maintain plasmid selection. Cultures were incubated anaerobically without shaking at 37°C until an optical density at 600 nm (OD_600_) of 0.4 was reached. To increase the amount of protein for purification purposes, the expression of the plasmid pASK-IBA3-borne *hypC* and *hybG* genes was induced by the addition of 0.2 μg ml^–1^ anhydrotetracycline (AHT), whereas for pT7-7- and pACYC-Duet1-based plasmids, gene expression was induced by the addition of 0.1 mM isopropyl β-D-1-thiogalactopyranoside (IPTG). Incubation of the cultures was continued at 30°C for 3 h, after which the cells were harvested by centrifugation at 5000 × *g* for 15 min at 4°C. The filling of bottles and tubes for the centrifugation of cultures and cell suspensions was performed under anoxic conditions in an anaerobic chamber (Coy Laboratories, Grass Lake, United States). Cell pellets, derived by centrifugation, were either used immediately or stored at – 20°C until use.

### Measurement of Hydrogen Production

Two methods were used to determine H_2_ production. One involved measuring cumulative H_2_ production after anaerobic cultivation of strains, while the other determined continuous H_2_ production in cell suspensions. Cumulative H_2_ content was determined by growing strains in 15 ml Hungate tubes (initially filled with N_2_) containing 8 ml of culture medium. The cultures were incubated for 20 h at 30°C and the H_2_ concentration was measured by removing 200 μl aliquots from the headspace and analyzing the gas-phase using gas chromatography with a GC2010 Plus Gas Chromatograph (Shimadzu, Kyõto, Japan) as described (Pinske et al., 2015). Pure nitrogen was used as the carrier gas, and the amount of H_2_ produced was calculated based on a standard curve prepared with pure H_2_ gas. The experiment was repeated three times and each assay was performed in triplicate.

Continuous H_2_ production by whole cells was determined using a modified Clark-type electrode equipped with an OXY/ECU module (Oxytherm, Hansatech Instruments, Norfolk, United Kingdom) to reverse the polarizing voltage to –0.7 V, essentially as described (Lindenstrauß et al., 2017). Cells were grown as described above, but only until the late-exponential phase was attained, and after the anaerobic centrifugation of cells to remove culture medium, the cell pellet was suspended in degassed 50 mM Tris, pH 7.0 and the centrifugation step was repeated. Subsequently, the cell pellet was suspended in 1 ml of degassed 50 mM Tris, pH 7.0 and 50 μl aliquots were added to the chamber of the electrode, which contained 1.95 ml of degassed 50 mM Tris, pH 7.0, equilibrated at 30°C. The reaction was started by adding 14 mM glucose, which was converted to formate intracellularly to act as a substrate of the FHL reaction and the amount of H_2_ produced was determined using pure H_2_ gas as described (Sargent et al., 1999). The assay was performed in triplicate using three biological replicates for each strain analyzed.

### Preparation of Crude Extracts for Determination of Hyd Enzyme Activity

Cell paste was suspended in 2 ml of 50 mM MOPS, pH 7, including 5 μg DNase I ml^–1^ and 0.2 mM phenylmethylsulfonyl fluoride per 1 g wet weight. Cells were disrupted by sonication (20 W for 2 min with 0.5 s pulses). Cell debris and unbroken cells were removed by centrifugation for 20 min at 21,000 × *g* at 4°C. The supernatant (crude extract) was carefully decanted into a fresh tube and was used immediately. Protein concentration was determined as described before (Lowry et al., 1951).

### Assay of Total H_2_-Oxidizing Hyd Enzyme Activity

The total Hyd enzyme activity of the crude extracts was determined as H_2_-dependent reduction of benzyl viologen (BV) as described (Ballantine and Boxer, 1985), except that the buffer used was 50 mM MOPS, pH 7.0. The wavelength used for the absorbance measurement was 600 nm and an ε_*M*_ value of 7,400 M^–1^ cm^–1^ was assumed for reduced BV. One unit of enzyme activity corresponded to the reduction of 1 μmol of substrate min^–1^. Enzyme assays were performed in triplicate using three biological replicates.

### Non-denaturing Polyacrylamide Gel Electrophoresis and Hyd Activity-Staining

Non-denaturing PAGE was performed according to Ballantine and Boxer (1985) using crude extracts (25 μg of protein). Prior to application onto the gel, crude extracts were incubated with a final concentration of 4% (v/v) Triton X-100 at 4°C for 15 min. Separating gels included 7.5% (w/v) polyacrylamide and 0.1% (w/v) Triton X-100. To visualize the activity of Hyd-1, Hyd-2, and Hyd-3, activity-staining after native PAGE was performed according to Pinske et al. (2012) using 50 mM MOPS, pH 7 buffer, which included 0.5 mM BV and 1 mM 2,3,5-triphenyltetrazolium chloride. Gels were incubated overnight at 25°C in an atmosphere of 9% N_2_: 5% H_2_. Experiments were repeated several times with the same results and a representative gel is shown.

### Protein Purification

All steps for protein purification were carried out in an anaerobic chamber (Coy Laboratories, Grass Lake, United States). Wet cell paste was suspended in 2 ml buffer W (50 mM Tris, pH 8, containing 150 mM NaCl) per 1 g cell paste. Phenylmethylsulfonyl fluoride (PMSF) was added to a final concentration of 0.8 mM and DNAse I to a final concentration of 10 μg/ml to the cell suspension. Cells were disrupted by sonication (Sonotrode, 35 W with 0.5 s pulses for 5 min) on ice. Cell debris and unbroken cells were removed by centrifugation at 21,000 × *g* for 20 min at 4°C. The supernatant obtained after centrifugation was used immediately for anaerobic protein purification. Strep-tag-II-tagged HypC and HybG were purified individually or in complex with HypD, using Strep-tactin sepharoseXT Sepharose (IBA Lifesciences, Göttingen), exactly as described (Soboh et al., 2013; Arlt et al., 2021). His-tagged pre-HycE was purified using cobalt-charged TALON Superflow agarose (Cytiva), following the manufacturer’s instructions.

Purified proteins were buffer-exchanged into anaerobic 50 mM Tris, pH 8, using 5 ml PD-10 columns containing G-25 matrix (Cytiva). The resulting protein samples were concentrated using Amicon centrifugal concentration filters (cut-off of 5 kDa for HypC and HybG proteins and 50 kDa for pre-HycE samples). Purified protein samples were stored at –80°C.

### Protein Interaction Studies Using Pull-Down Assays and Western Blotting

To examine the interaction between Strep-tagged HybG_*WT*_, HybG_*H*52*A*_, HypC_*WT*_, or HypC_*H*51*A*_ with His-tagged pre-HycE 150 μg of each protein (5:1 mol excess of chaperone) was mixed and incubated at 30°C under anoxic conditions for 2 h. After incubation, the mixture was loaded onto either a 0.5 ml Strep-tactin sepharoseXT column or a 0.5 ml cobalt-charged TALON Superflow agarose column for the enrichment of the interaction partners. Columns were pre-equilibrated with buffer W (50 mM Tris/HCl, pH 8, containing 150 mM NaCl). After loading of sample, the columns were washed with 10 column volumes of buffer W to remove unbound proteins. Bound proteins were subsequently eluted with buffer W containing 50 mM biotin (Strep-tactin sepharoseXT columns), or with buffer W containing 300 mM imidazole (cobalt-charged NTA columns). Fractions of 0.5 ml were collected and aliquots from these were analyzed by electrophoresis on 12.5% (w/v) or 1% (w/v) denaturing polyacrylamide sodium dodecylsulfate (SDS)-PAGE (Laemmli, 1970). After the separation of polypeptides, they were transferred onto a nitrocellulose membrane as described (Towbin et al., 1979). After blocking the membrane, interaction partners were identified by challenging with polyclonal antiserum raised against HypC, HybG, or HycE (Pinske et al., 2015; Arlt et al., 2021). The detection was based on chemiluminescence using the Immuno-detection kit SuperSignal West Pico PLUS (Thermo Scientific, Brunswick, Germany) and an imager Amersham Imager 600 (GE Healthcare Bio-Sciences AB, Solingen, Germany).

## Results

### An H52A-Exchange in HybG Results in Wild-Type Levels of H_2_ Production

The HypC chaperone preferentially matures pre-HycE, the Hyd-3 large subunit precursor, while its paralogue HybG preferentially introduces the Fe(CN)_2_CO group of the [NiFe]-cofactor into pre-HyaB and pre-HybC, the respective precursors of Hyd-1 and Hyd-2 (Blokesch et al., 2001; Arlt et al., 2021). As the aim of this study was to determine the significance of the conserved His residue in HypC and HybG for their function, we decided to exchange the large, charged histidine residue in both proteins for a small, non-polar alanine residue. First, a series of eight plasmids carrying either the native *hypC* gene, the native *hybG* gene, or carrying *hypC* + *hypD*, or *hybG* + *hypD* together, was constructed (Table 1). Derivatives of these four plasmids were also constructed, in which codon 51 in *hypC* and codon 52 in *hybG* were mutated to decode as an alanine residue (see section “Materials and Methods”). It is important to note that in all experiments described in the current study, HypC and HybG both carried a *C*-terminal Strep-tag II. The presence of this StrepII-tag does not interfere with the functionality of either protein with respect to Hyd precursor maturation (Blokesch et al., 2004; Soboh et al., 2013; Thomas et al., 2018). Consequently, in the interest of convenience, we will henceforth generally refer to these proteins without mentioning the tag.

**FIGURE 2:**
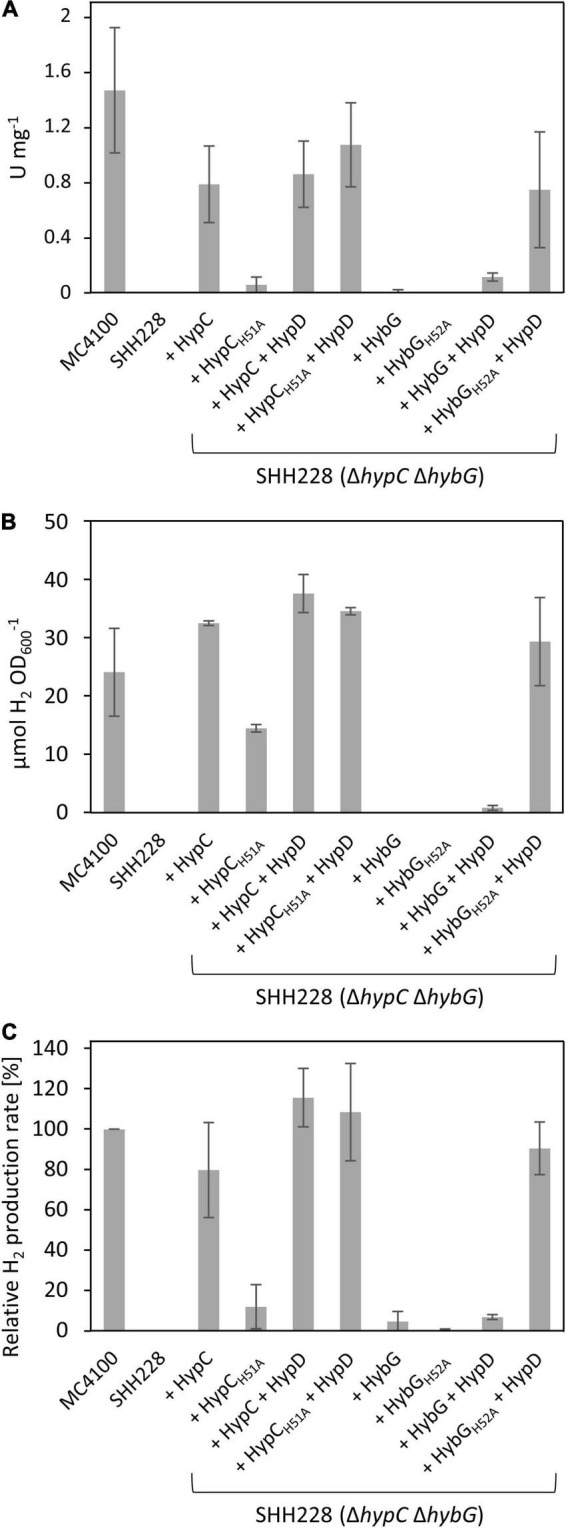
H_2_-oxidizing and H_2_-evolving activities of *E. coli* strain SHH228 (Δ*hypC*Δ*hybG*) synthesizing different HypC and HybG variants. **(A)** Total H_2_-oxidizing hydrogenase enzyme activity, measured as H_2_-dependent BV reduction (see section “Materials and Methods”) was determined in crude extracts derived from anaerobically grown SHH228 transformed with the following plasmids: phypCstrep, (HypC); phypC(H51A)strep, (HypC_*H*51*A*_); pT-hypDCStrep, (HypD + HypC); pT-hypDC(H51A)Strep, (HypD + HypC_*H*51*A*_); phybGstrep, (HybG); phybG(H52A)strep, (HybG_*H*52*A*_); pT-hybG-hypDEF, (HypD + HybG); pT-hybG(H52A)-hypDEF, (HypD + HybG_*H*52*A*_). MC4100 is the isogenic parental wild-type strain of SHH228 (Δ*hypC*Δ*hybG*). One unit of enzyme activity corresponded to the reduction of 1 μmol of substrate min^– 1^. **(B)** The total accumulated H_2_ production after fermentative growth of the same strains as shown in part **(A)** was determined after 20 h of anaerobic growth in TGYEP, pH 6.5 (see section “Materials and Methods” for details). The amount of H_2_ in an aliquot of 200 μl of the gas phase was determined. **(C)** H_2_ evolution rates in freshly harvested exponentially growing cells were determined for the same strains as in parts **(A,B)** using whole cells and glucose as reductants (see section “Materials and Methods” for details). The data are presented as a percentage relative to the activity determined for MC4100, which was 46.3 ± 8.3 nmol H_2_ min^– 1^ mg^– 1^ and represented the 100% value. All assays **(A–C)** show data as standard deviations from the mean, determined using at least three independent biological replicates, each assayed in duplicate or triplicate.

As the first experiment to examine the potential effects of exchanging the conserved histidine residues in HypC and HybG to alanine on the maturation of the Hyd-1, -2, and -3 enzymes, we first determined the total H_2_-oxidizing Hyd enzyme activity (Figure 2A). Therefore, strain SHH228 (see Table 1), lacking genomic copies of both *hypC* and *hybG*, but retaining *hybD* (Hartwig et al., 2015), was transformed with each of the eight plasmids individually. After the anaerobic growth of the strains and preparation of crude extracts (see section “Materials and Methods” for details), the total Hyd enzyme activity was determined for each (Figure 2A). An extract derived from the parental strain MC4100 had a total Hyd activity of approximately 1.5 U mg^–1^ and served as a positive control, while an extract derived from SHH228 (*hypC hybG*) had no detectable activity and acted as the negative control (Figure 2A). The introduction of plasmid phypCstrep carrying the parental *hypC* gene restored approximately 50% of the parental total Hyd enzyme activity to the mutant. This is consistent with HypC being required for the maturation of Hyd-3 and with the fact that under these growth conditions, Hyd-3 constitutes the bulk of the total Hyd activity (Sawers et al., 1985; Pinske et al., 2011). In contrast, the isogenic plasmid phypC(H51A)strep with a mutation in codon 51 of *hypC* only restored the total Hyd activity to a level that was less than 5% of that measured for the positive control MC4100 (Figure 2A). This indicates that the H51A amino acid exchange in HypC severely compromised its ability to function in maturation of Hyd-3, which is also consistent with the reduced amount of Fe(CN)_2_CO group detected after purification of HypD associated with an HypC_*H*51*R*_ variant reported earlier (Soboh et al., 2013).

As the *hypC* gene is typically found located adjacent to *hypD* in the genomes of microorganisms that synthesize [NiFe]-Hyd, and because HypC and HypD form the central scaffold complex during the maturation of these enzymes (Böck et al., 2006; Lacasse and Zamble, 2016), we wished to determine whether their multicopy co-expression might rescue the Hyd-deficient phenotype exhibited by SHH228 synthesizing HypC_*H*51*A*_, despite this strain already possessing a genomic copy of *hypD*. Therefore, we determined the total Hyd enzyme activity of the strain co-expressing both genes from the same plasmid. When the native *hypC* gene was co-expressed with *hypD*, a similar total Hyd enzyme activity was determined compared to when *hypC* was expressed alone from plasmid phypCstrep (Figure 2A). When the same experiment was repeated using a plasmid co-expressing *hypD* (pT-hypDCstrep in Table 1) and the mutated *hypC* gene synthesizing HypC_*H*51*A*_ [pT-hypDC(H51A)strep, Table 1], the total Hyd enzyme activity was recovered to a level close to that measured for the wild-type strain. This result suggests that HypC_*H*51*A*_ is still functional in the maturation of Hyd-3, but is less efficient at completing maturation unless HypD and the HypC proteins are co-over-produced.

The *hybG* gene, encoding HybG, is located within the *hyb* operon (Menon et al., 1994) and does not have its own associated *hypD* gene. The introduction of a plasmid carrying only *hybG*, encoding Strep-tagged native HybG, into strain SHH228 (Δ*hypC* Δ*hybG*) failed to result in restoration of wild-type total Hyd activity to the mutant and activity was barely detectable (Figure 2A). It should be noted that the percentage contribution of Hyd-1 plus Hyd-2 activity to the total H_2_-oxidizing Hyd activity under fermentative conditions is only between 10–20% (Sawers et al., 1985; Pinske et al., 2011). This does not, however, explain the poor complementation achieved by re-introducing plasmid-borne native *hybG*. Therefore, to determine whether the co-expression of *hybG* with *hypD* might improve complementation and increase H_2_-oxidizing Hyd activity, plasmid pT-hybG-hypDEF was tested. After fermentative growth, crude extracts derived from SHH228/pT-hybG-hypDEF revealed an increase in the total Hyd activity to around 10% of the total activity measured for the wild-type MC4100 (Figure 2A), which approximately represents an activity consistent with that expected for Hyd-1 plus Hyd-2 under these growth conditions (Pinske et al., 2011). Note that plasmid pT-hybG-hypDEF also carries the *hypE* and *hypF* genes. The presence of these genes does not affect the total Hyd enzyme activity determined compared to when only *hybG* + *hypD* or only *hypC* + *hypD* is present on the plasmid (Haase and Sawers, unpublished results).

The anticipated transformation of SHH288 with plasmid phybG(H52A)strep, which has a mutation in codon 52 in the *hybG* gene resulting in exchange of histidine for alanine and delivering HybG_*H*52*A*_, failed to yield measurable Hyd activity (Figure 2A). Surprisingly, however, when *hypD* was co-expressed with this mutated *hybG* gene on pT-hybG(H52A)-hypDEF, the total Hyd activity measured was similar to that measured for *hypC-hypD* on pT-hypDCStrep in SHH228 (Figure 2A). This result suggests that this approximate 10-fold increase in total Hyd activity, relative to what was measured when pT-hybG-hypDEF was introduced into SHH228, was either due to an unexpected increase in the combined Hyd-1 and Hyd-2 activity, or because Hyd-3 activity was activated by the variant HybG_*H*52*A*_ protein when it was co-over-produced with HypD.

### An H52A Exchange in HybG Restores H_2_ Production to a *hypC-hybG Escherichia coli* Mutant

In order to resolve this issue, we first tested the same set of strains for their ability to produce H_2_ during glucose fermentation (Figures 2B,C). The amount of H_2_ accumulated in stationary-phase cells after batch cultivation revealed that the positive control MC4100 accumulated approximately 25 μmol H_2_ OD_600_ nm^–1^, while the negative control SHH228 (Δ*hypC* Δ*hybG*) accumulated no detectable H_2_ gas (Figure 2B). SHH228 transformed with pT-hybG(H52A)-hypDEF (synthesizing HypD plus HybG_*H*52*A*_) accumulated H_2_ to a level that was slightly more than that of the parental MC4100 strain, indicating that Hyd-3 was active. The same experiment performed with a plasmid bearing *hypD* and the parental *hybG* genes (pT-hybG-hypDEF) showed essentially no H_2_ accumulation, as did SHH228 transformed with plasmids carrying only the parental *hybG* or only the mutated *hybG* genes lacking the additional *hypD* gene (Figure 2B). This supports the conclusion that the mutant HybG_*H*52*A*_ chaperone, synthesized at a high level together with HypD, was capable of maturing Hyd-3 and thus accounted for the H_2_ production by the strain. As further controls, when SHH228 co-over-produced either HypC or HypC_*H*51*A*_ together with HypD, H_2_ also accumulated to wild-type levels (Figure 2B). However, when the strain only synthesized HypC_*H*51*A*_, H_2_ accumulated to approximately 50% of parental levels.

To verify these results, the ability of the same set of strains to evolve H_2_ in cell suspensions derived from exponentially grown cells was assessed using a hydrogen-electrode (Figure 2C). The results essentially reflected those obtained by measuring cumulative H_2_ production, with the exception that SHH228 synthesizing HypC_*H*51*A*_ from phypC(H51A)strep had an H_2_-evolving activity that was only 10% of that of the wild-type strain MC4100 (Figure 2C). These results confirmed the poor complementation of the Hyd -deficient phenotype exhibited by this strain when the total Hyd activity was measured in extracts (compare Figure 2A). It is likely that in the cumulative H_2_ assay, the cells had sufficient time to accumulate H_2_ in the stationary phase, which probably accounts for the difference when the two assay methods for H_2_ production by the strain are compared (compare Figures 2B,C).

### The HybG_*H*52*A*_ Variant Is Able to Mature Hyd 3

**FIGURE 3:**
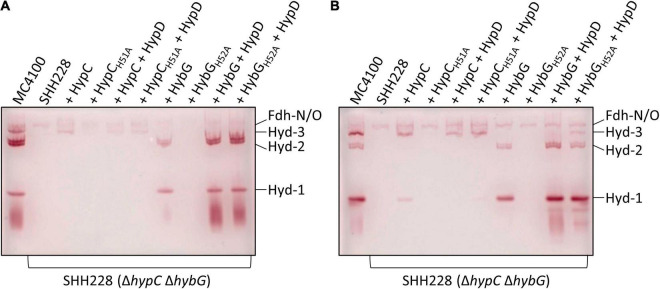
Identification of hydrogenases 1, 2, and 3 by enzyme activity-staining. Protein complexes in crude extracts (25 μg of protein) derived from the indicated strains (see legend to Figure 1A) were separated in native polyacrylamide gel electrophoresis (PAGE) (7.5% w/v polyacrylamide) and subsequently stained for hydrogenase enzyme activity (see section “Materials and Methods”). Cells were grown anaerobically in TGYEP, pH 6.5, and harvested in the exponential phase (**A**; cultivation to OD_600_ 0.7) or in the stationary phase (**B**; cultivation at 30°C for 16 h). The migration positions of Hyd-1, Hyd-2, and Hyd-3 are shown on the right side of the gels. The activity-staining band labeled Fdh-N/O signifies the weak H_2_-oxidizing activity associated with the formate dehydrogenases N and O and acted as a loading control.

Bands corresponding to Hyd-1, Hyd-2, and Hyd-3 can be readily distinguished after native PAGE followed by staining the gel specifically for Hyd enzyme activity (Pinske et al., 2012). Moreover, this technique also allows the identification of an H_2_:benzyl viologen oxidoreductase activity associated with the formate dehydrogenases (Fdh) N and O, which is a side-reaction of these enzymes (Soboh et al., 2011), but is a useful control for these experiments. As HybG typically cannot facilitate the maturation of Hyd-3 (Blokesch et al., 2001; Böck et al., 2006), we first looked at the extracts derived from both exponential-phase (Figure 3A) and stationary-phase cells (Figure 3B) for evidence of HybG-dependent synthesis of active Hyd-3. Regardless of whether the native *hybG* gene was expressed alone, or co-expressed with *hypD* from a plasmid, no manifestation of active Hyd-3 after native PAGE and staining for Hyd enzyme activity could be observed (Figure 3). In contrast, when HybG_*H*52*A*_ was co-synthesized with HypD in strain SHH288 (Δ*hypC* Δ*hybG*), and in the absence of any HypC, Hyd-3 activity could be visualized (Figure 3B). Although the activity band was relatively weak, it was clearly visible, especially in the crude extract derived from stationary-phase cells. Moreover, the activity band had an intensity comparable to that in exponential-phase cells when the native *hypC* gene was expressed on its own from plasmid phypCstrep (Figure 3A). This result indicates that HybG_*H*52*A*_ can mature the large subunit precursor (pre-HycE) of Hyd-3.

Hydrogenase-1 is more active in anaerobic stationary-phase cells, while Hyd-2 is more active in exponential-phase cells (Ballantine and Boxer, 1985; Sawers et al., 1985). The examination of the activity-stained bands in the other extracts revealed that, although Strep-tagged native HybG was capable of restoring maturation of both Hyd-1 and Hyd-2 in exponential- and stationary-phase cells (Figures 3A,B), the intensity of the respective activity bands was low. This possibly explains the poor phenotypic complementation by plasmid-borne *hybG* when introduced into SHH228 and the very low total Hyd activity measured in Figure 2A. Strain SHH228, synthesizing the HybG_*H*52*A*_ variant, showed no activity bands corresponding to either Hyd-1 or Hyd-2 when the cognate gene was expressed on its own from plasmid phybG(H52A)strep (Figures 3A,B). Notably, however, when either *hybG* allele was co-expressed with *hypD*, extracts derived from the corresponding cells revealed wild-type levels of activity-staining bands for Hyd-1 and Hyd-2 (Figure 3). These results suggest that the co-synthesis of HypD either resulted in the stabilization of the respective HybG chaperones when the cognate genes were co-expressed or facilitated interaction of the proteins to allow scaffold complex formation, thus improving the efficiency of maturation of both the large-subunit precursors, pre-HyaB and pre-HybC.

In this regard, crude extracts derived from stationary-phase cells of SHH288/phypCstrep, which synthesized Strep-tagged native HypC, revealed only a weak activity band that migrated at the position of Hyd-1, but no such similar activity band was observed in an extract derived from SHH288 co-expressing *hypC* and *hypD* (Figure 3B); the levels of Hyd-3 remained similar for both strains, providing an internal loading control. This underscores the preferential maturation of pre-HycE over pre-HyaB by HypC carrying Fe(CN)_2_CO (see also Figure 1A).

### HybG_*H*52*A*_ Interacts Better With Pre-HycE but Worse With HypD

**FIGURE 4:**
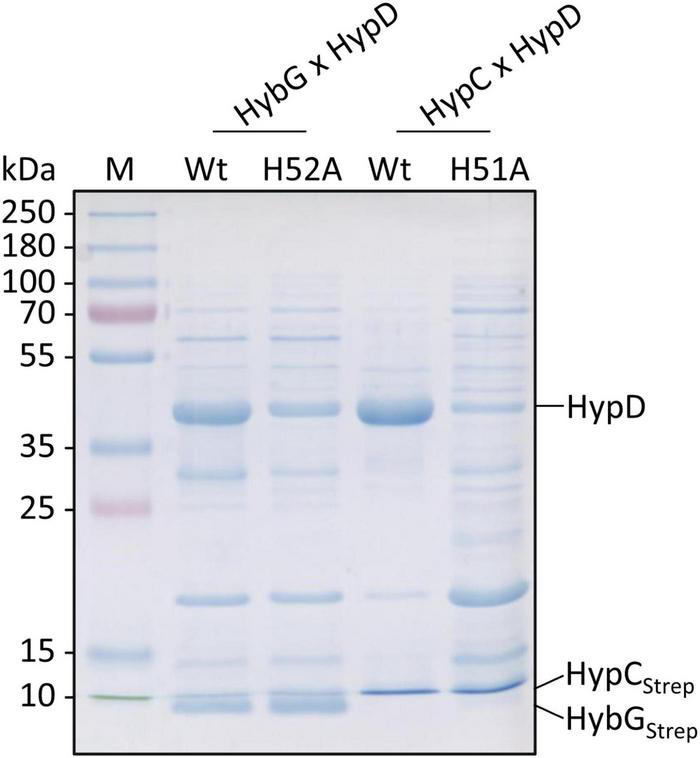
Analysis of co-enrichment of untagged HypD with Strep-tagged HypC and HybG variants. Aliquots of elution fractions (10 μg protein) were separated by SDS-PAGE (12.5% w/v acrylamide) followed by staining with Coomassie Brilliant Blue. The migration positions of HypD, Strep-tagged HypC (HypC_*Strep*_), and HybG (HybG_*Strep*_) are indicated at the right side of the panel. Migration positions of molecular mass markers are shown on the left of the gel.

HypC-HypD and HybG-HypD complexes can be readily isolated from anaerobically cultivated cells (Blokesch et al., 2004; Soboh et al., 2012) and it has been shown using structural analyses (Watanabe et al., 2012) and by native mass spectrometry (Arlt et al., 2021) that both sets of complexes form (1:1) heterodimers. To determine whether the exchange of the conserved histidine residue that is predicted to be important for the interaction with HypD (see Figure 1A; Watanabe et al., 2012; Miki et al., 2020) has an impact on this interaction, we enriched, by affinity chromatography in a single step HybG-HypD, HybG_*H*52*A*_-HypD, HypC-HypD, and HypC_*H*51*A*_-HypD complexes from anaerobically grown SHH228 cells. To do this, we took advantage of the StrepII-tag on the chaperones (see section “Materials and Methods”). The resulting complexes were separated by SDS-PAGE and visualized using Coomassie Blue staining (Figure 4). While the native HybG and HypC proteins each could be enriched together with high amounts of HypD and minimal contaminating polypeptides, the mutated chaperone proteins clearly interacted more poorly with HypD. The complexes formed were less-well resolved, the affinity-enriched samples included considerably more contaminating proteins, and, based on densitometric analysis (ImageQuant TL program Cytiva), the apparent stoichiometries were significantly lower than those observed for the respective native chaperone (at least 50% lower for HybG_*H*52*A*_:HypD and ∼85% lower for HypC_*H*51*A*_:HypD).

**FIGURE 5:**
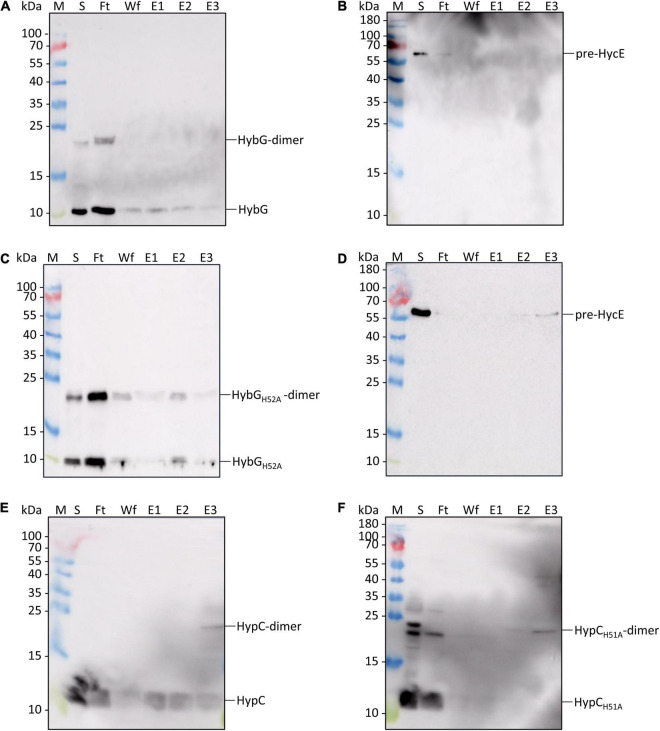
Identification of interaction partners: HybG_*H*52*A*_ forms a complex with pre-HycE. Purified Strep-tagged HypC_*WT*_, HypC_*H*51*A*_, HybG_*WT*_, or HybG_*H*52*A*_ was mixed with purified His-tagged pre-HycE and after affinity chromatography, aliquots of the original sample mixture (S), the unbound flow-through (Ft), the material washed from the column (Wf) and the eluted material (fractions E1, E2, and E3) were separated by SDS PAGE followed by western blotting. Membranes were treated with antiserum raised against HypC, HybG, or HycE, each diluted 1:4000. **(A)** Blot treated with anti-HybG antiserum after separation of a mixture of Strep-tagged HybG and His-tagged pre-HycE on a cobalt-charged TALON agarose column. HybG monomers and dimers (dimers are occasionally observed after SDS-PAGE; Arlt et al., 2021) are indicated on the right of the blot. Migration positions of molecular mass markers are shown on the left of the blot. **(B)** The same experiment as shown in panel **(A)** was performed, but after the separation of the mixture on a Strep-tactin sepharose column, His-tagged pre-HycE was detected with anti-HycE antiserum. **(C)** A mixture of purified Strep-tagged HybG_*H*52*A*_ and pre-HycE was separated on a cobalt-charged TALON agarose column as described in part **(A)**. HybG_*H*52*A*_ was detected with anti-HybG antiserum. **(D)** The same mixture as in part **(C)** was separated on a Strep-tactin sepharose column and pre-HycE was detected with anti-HycE antiserum. **(E)** Western blot in which a mixture of Strep-tagged HypC and His-tagged pre-HycE was separated on a cobalt-charged TALON agarose column and HypC was detected with anti-HypC antiserum. **(F)** Western blot in which a mixture of Strep-tagged HypC_*H*51*A*_ and His-tagged pre-HycE was separated on a cobalt-charged TALON agarose column, with subsequent detection using anti-HypC antiserum.

Next, we purified separately Strep-tagged native HybG and HybG_*H*52*A*_, as well as a His-tagged derivative of pre-HycE, the precursor of the Hyd-3 large subunit. These were then used in interactions experiments (see section “Materials and Methods”) to test whether HybG_*H*52*A*_ could form a complex with pre-HycE (Figure 5). The results show that when complexes were allowed to form between His-tagged pre-HycE and Strep-tagged native HybG and the mixture was subsequently separated on a cobalt-charged TALON Superflow agarose column, only low amounts of HybG co-eluted with pre-HycE (Figure 5A). When the same mixture was passed over a Strep-tactin sepharose column, no pre-HycE could be identified to co-elute with Strep-tagged HybG (Figure 5B). In contrast, when the same experiment was repeated with Strep-tagged HybG_*H*52*A*_, more of the chaperone was shown to co-elute with His-tagged pre-HycE after cobalt-charged TALON agarose chromatography (Figure 5C), and pre-HycE could clearly be identified to co-elute with Strep-tagged HybG_*H*52*A*_ after Strep-tactin sepharose affinity chromatography (Figure 5D).

In a similar experiment performed with Strep-tagged HypC, Strep-tagged HypC_*H*51*A*_, and His-tagged pre-HycE, native HypC co-eluted strongly with His-tagged pre-HycE (Figure 5E), while HypC_*H*51*A*_ interacted more poorly with pre-HycE (Figure 5F).

## Discussion

In *E. coli*, the scaffold protein HypD interacts with two distinct but related HypC-family chaperones, which in turn deliver the synthesized Fe(CN)_2_CO group to three separate large-subunit precursors. Although both chaperones interact (with different effectiveness) with pre-HyaB, the Hyd-1 large-subunit precursor, only HypC delivers the iron group to pre-HycE, while pre-HybC receives the group exclusively from HybG (Blokesch et al., 2001; Arlt et al., 2021). Here, we have resolved two key discriminatory functions associated with a conserved histidine residue within the HypC-family chaperone member, HybG. Although only semi-quantitative, the results of our interaction experiments give a first clear indication that an H52A residue exchange in HybG diminishes the ability of the chaperone to interact with its scaffold partner HypD. This result was corroborated when a similar exchange in HypC was made, which also resulted in a diminished interaction with HypD when compared with native HypC. An H-to-A residue exchange in either protein thus appears to weaken the interaction with the scaffold protein HypD, with which it functions to synthesize the Fe(CN)_2_CO group (Böck et al., 2006). This also provides a biochemical explanation for the previous observation that an exchange of H51 for an arginine residue in HypC impaired Hyd-3-dependent H_2_ evolution by *E. coli* (Blokesch, 2004; Soboh et al., 2013). Based on the structural analysis of the HypC-HypD complex (see Figure 1B), this histidine residue is predicted to form part of the contact surface between HybG and HypD but has also been suggested possibly to aid the coordination of the Fe(CN)_2_CO group when the chaperone delivers the group to the large-subunit precursor (Watanabe et al., 2007, 2012). The findings made in the current study are consistent with this residue being important for the interaction with HypD but do not support a role for the residue in coordinating the organometallic iron group, because our experiments involving co-synthesis of either HypC_*H*51*A*_ or HybG_*H*52*A*_ with HypD revealed that efficient maturation of their cognate large-subunit precursors still occurred with both chaperone variants. For example, the observed reduction in Hyd-2 activity caused by the H52A exchange in HybG could be totally restored if the gene encoding HybG_*H*52*A*_ was co-expressed with *hypD*. This suggests that the interaction between HybG_*H*52*A*_ [bearing the Fe(CN)_2_CO group] and the pre-HybC large-subunit precursor is not strongly compromised by the exchange, and the observed reduction in Hyd-2 activity might be due simply to the poorer interaction of HybG_*H*52*A*_ with HypD. Nevertheless, future quantitative assessment of binding affinities for these various interaction partners will be needed to verify this proposal.

This contrasts sharply, however, with the demonstration of improved interaction between HybG_*H*52*A*_ and pre-HycE, which resulted in an unprecedented HybG-dependent maturation of Hyd-3. This result suggests that the histidine residue functions to prevent HybG from interacting with pre-HycE. When histidine is substituted by the much smaller alanine residue, this inhibition is relieved, allowing interaction and maturation to take place. A similar residue exchange in HypC (H51A) did not have any effect on Hyd-2 enzyme activity, only causing decreased H_2_ production, because pre-HycE is HypC’s principal interaction partner (Drapal and Böck, 1998; Blokesch et al., 2004; Böck et al., 2006). Thus, the HypC_*H*51*A*_ variant does not become capable of interacting with pre-HybC to generate active Hyd-2, suggesting that pre-HycE has structural features at its chaperone interaction surface that differ from those of both pre-HybC and pre-HyaB. Future structural comparisons of these precursors will be highly beneficial, coupled with a chemical cross-linking analysis, to define these interacting residues.

Thus, two features of the HybG chaperone seem to be important in ensuring it only matures the H_2_-oxidizing Hyd-1 and Hyd-2 enzymes *in vivo*. Firstly, expression of the *hybG* gene within the *hyb* operon, plus its physical separation from the *hypD* gene, ensures that HybG and pre-HybC are synthesized together. Nevertheless, HybG is produced in sufficient amounts also to allow maturation of pre-HyaB (precursor of Hyd-1) as well as pre-HybC, and it appears to be more effective at this than HypC (see also Blokesch et al., 2001). The fact that co-expressing the *hypD* gene with the mutated *hybG* gene improved complementation ability may be due to a form of translational coupling that compensates for the poorer HypD-HybG_*H*52*A*_ complex formation by improving their chances of interacting in the cell. The consequence is that sufficient production of the Fe(CN)_2_CO group occurs to facilitate the maturation of all three Hyd large subunit precursors.

Secondly, the H52 residue, as well as being required for optimal interaction with HypD, acts to prevent HybG from interacting with pre-HycE. Non-native co-overexpression of the native *hybG* and *hypD* genes still failed to result in inadvertent maturation of pre-HycE, but not when codon 52 of *hybG* coded for an alanine residue. The selective maintenance of H52 in HybG thus assures effective HybG-HypD scaffold complex formation, while at the same time guaranteeing that pre-HycE cannot be matured by HybG. This study thus identifies a new layer of control during Hyd maturation. Controlling which chaperone (HypC or HybG) preferentially interacts with HypD determines which Hyd precursor is matured in accordance with the demands set by the physiological conditions. This ultimately results in the balanced production of H_2_-evolving and H_2_-oxidizing enzyme activities.

## Data Availability Statement

The raw data supporting the conclusions of this article will be made available by the authors, without undue reservation.

## Author Contributions

AH and RGS conceived and designed the study, analyzed the data, and drafted the manuscript. AH performed all of the experiments. Both authors approved the manuscript.

## Conflict of Interest

The authors declare that the research was conducted in the absence of any commercial or financial relationships that could be construed as a potential conflict of interest.

## Publisher’s Note

All claims expressed in this article are solely those of the authors and do not necessarily represent those of their affiliated organizations, or those of the publisher, the editors and the reviewers. Any product that may be evaluated in this article, or claim that may be made by its manufacturer, is not guaranteed or endorsed by the publisher.
